# A Novel Nomogram Combining Alternative Splicing Events and Clinical Factors for Prognosis Prediction in Head and Neck Squamous Cell Carcinoma

**DOI:** 10.1155/2022/4552445

**Published:** 2022-01-22

**Authors:** Jun Jiang, Li Niu, Ming-Xia Zhang, Hao Wang, Jia-Qi Xie, Guo-Ping Sun

**Affiliations:** ^1^Department of Radiation Oncology, The First Affiliated Hospital of Anhui Medical University, Hefei, Anhui, China; ^2^Department of Cancer Center, Drum Tower Hospital of Nanjing University, Nanjing, Jiangsu, China; ^3^Department of Medical Oncology, The First Affiliated Hospital of Anhui Medical University, Hefei, Anhui, China

## Abstract

Due to limitations of sensitive biomarkers, the clinical prognosis of patients with head and neck squamous cell carcinoma (HNSCC) remains poor. Alternative splicing (AS) is the basis of both transcriptome and proteome richness, so more and more evidence indicates an important relationship between AS and tumor progression. The aim of this study was to offer a comprehensive analysis on AS events and then investigate its potentials as a new biomarker for patients with squamous cell carcinoma of the head and neck. In this study, univariate assays were conducted to examine the prognosis-associated AS events, and we screened 4068 survival-related AS events in 2573 genes. Then, the AS events related to survival were further determined and analyzed using LASSO regression and multivariate assays, and an eleven-AS signature was developed. Kaplan–Meier assays indicated patients with high-risk scores exhibited a shorter OS than those with low-risk scores. Multivariate assays further demonstrated that the signature's risk score was independent of HNSCC survivals. Meanwhile, we analyzed the clinical association of AS-based prognostic signature in HNSCC patients and observed that tumor specimens with advanced stages and grades exhibited a high risk score. In addition, the results of survival nomogram revealed that predicted outcomes and actual outcomes were highly consistent. Overall, our group showed an eleven-AS signature of HNSCC, which could be regarded as a separate prognostic factor.

## 1. Introduction

Head and neck squamous cell carcinoma (HNSCC) is the most common malignant tumor of the epidermis of the head and neck, involving multiple anatomical sites, such as the lip, oral cavity, pharynx (nasopharynx, oropharynx, and hypopharynx), and larynx, and more than 600,000 new cases are reported every year [1, 2]. Despite the distinct developments in molecular mechanisms and biological studies, the long-term survivals of patients with HNSCC remain poor [3]. Thus, a suitable choice for different patients using radical treatments or conservative treatments is necessary. In the last twenty years, the prediction of clinical outcome of HNSCC patients was mainly based on the TNM staging system [4]. In addition, differentiation grade is also applied as a critical predictor. However, these systems cannot satisfy clinical requirements.

Alternative splicing (AS) is considered to be a critical impetus for the production of different types of proteins [5]. In eukaryotic cells, it is the basis for the other regulatory mechanisms involved in gene functions. A wealth of supporting evidence has indicated that transcripts >95% of human multiexon-containing genes experience AS [6]. Importantly, based on the different types of specimen, a variable expression was observed in most genes [7]. It has been confirmed that there are seven major patterns of AS events, including mutually exclusive exons (MEs), alternate terminator (AT), alternate promoter (AP), alternate acceptor site (AA), alternate donor site (AD), and retained intron (RI), as well as exon skip (ES) [8, 9]. The dysregulation of AS events could result in multiple pathological processes, especially tumor progression and chemotherapy resistance. Splicing factors (SFs) exhibited a critical role in the progression of various tumors induced by AS [10, 11]. More importantly, the potential of AS events used as novel biomarkers for diagnosis and prognosis attracts more and more attention [12, 13]. On the other hand, targeting AS events may be developed as novel therapeutic targets for tumor patients.

The clinical data from TCGA datasets made the analysis of AS in cancers possible. Recently, a large number of studies have performed comprehensive analysis based on TCGA splicing data in several types of tumors [14, 15]. However, there are very few reports on the correlation between AS events and the clinical outcomes of HNSCC patients. In this study, a comprehensive analysis was performed by using TCGA datasets to discuss the prognostic value of AS events in patients with HNSCC. Our findings may contribute to the developments of novel biomarkers for tumor patients.

## 2. Materials and Methods

### 2.1. Data Acquisition and Processing

There were 546 samples in FPKM data of TCGA RNA-Seq that were downloaded from the UCSC Cancer Browser (https://xenabrowser.net/datapages/), and a total of 528 patients were followed up. The alternative splicing data of the TCGA HNSCC cohort were downloaded from the TCGASpliceSeq database (https://bioinformatics.mdanderson.org/TCGASpliceSeq/). The same TCGA ID was applied to confirm the data of AS events.

### 2.2. Quantification of Splicing Events

Percent Spliced In (PSI) values were calculated in all samples. The PSI values (>0 and <100%) represented the percentage of gene mRNA transcripts that contain a specific exon or splice. Here, an AS event whose PSI value was larger than 75% was included for further assays. The AS events were exhibited by the use of three elements.

### 2.3. Identification of AS Events Related to Survival

For the survival assays, our group just finally enrolled these patients who had AS event data and clinical follow-up. In addition, HNSCC patients whose survival time <1 month were excluded. After excluding AS events with SD <0.01, univariate assays were conducted to examine the associations between each AS event and overall survival in HNSCC patients. Then, the correlation between AS events and genes was visualized by the use of UpSet [16].

### 2.4. Prognostic Model Construction

To screen the final AS events for prognostic model, the OS-related AS events were analyzed by using lasso analysis. Then, multivariate assays were applied to analyze the results of lasso analysis via the forward stepwise methods. Subsequently, by the use of each prognostic model, we calculated risk scores, and the median risk score was applied to divide all patients into two groups. The predictive accuracy of the prognostic models was demonstrated using dynamic time-dependent ROC curves and K–M survival assays. To realize the abovementioned assays, we used timeROC package, survivalROC package, and the survminer package.

### 2.5. AS-Clinicopathological Nomogram

To further explore the prognostic value of the prognostic model, univariate assays were applied to analyze the clinicopathological variables described above with the prognostic models. Then, a nomogram was developed by the use of the abovementioned results with a distinct *p* value to examine the patients' individual survival possibilities. Finally, corresponding calibration curves were plotted, which were further used to calculate the C-index and validate and quantify the scoring system's discrimination capability.

### 2.6. Statistical Analysis

We used R (v.3.6.1, R Core Team, Boston, MA, USA) for the abovementioned data analysis.

## 3. Results

### 3.1. Details of AS Events

**Figure 1:**
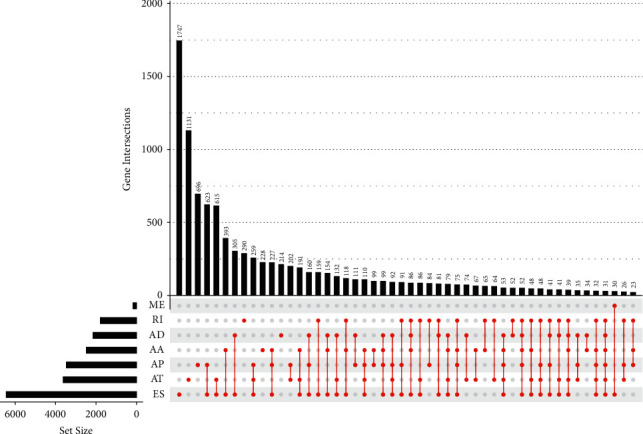
The upset plot of gene interactions among the seven types of AS events in HNSCC samples.

By analyzing TCGA datasets, we showed 42849 AS events of 10123 genes in all samples.  Figure 1 exhibited the detailed information of the seven categories of AS events. We observed that a single gene could possess some different AS patterns.

### 3.2. Identification of the OS-Associated AS Events

**Figure 2:**
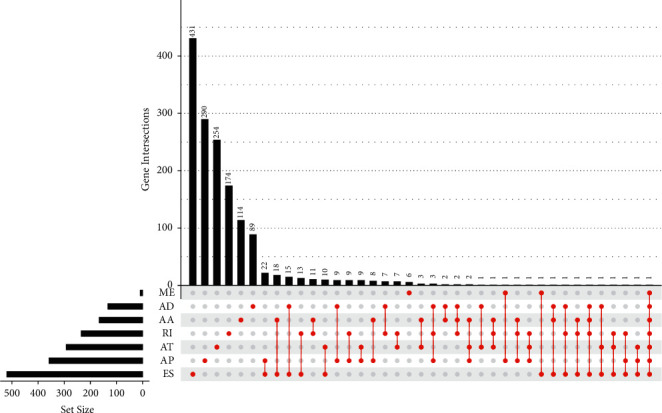
The upset plot of gene interactions based on the survival-associated AS events.

**Figure 3:**
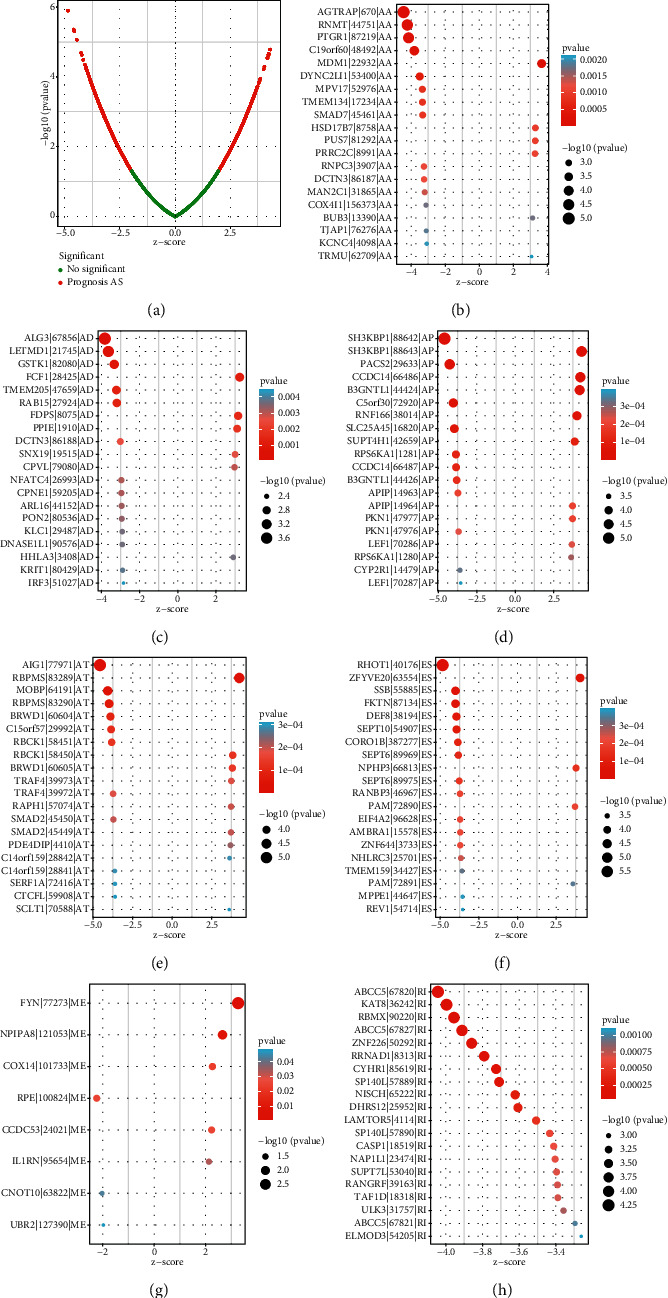
The survival-associated AS events. (a) Survival-related AS events were displayed by the use of a volcano plot. (b–h) The most distinct prognosis-related AA, AD, AP, AT, ES, ME, and RI in TCGA HNSCC datasets.

Univariate assays were performed, and 4068 OS-related AS events were *screened* in 2573 genes. Of the OS-related AS events, 276 OS-related RIs were found in 235 genes, 14 OS-related MEs in 9 genes, 608 OS-related ESs in 519 genes, 522 OS-related ATs in 292 genes, 588 OS-related APs in 358 genes, 140 OS-related ADs in 133 genes, and 169 OS-related AAs in 166 genes (Figure 2). The distribution of the OS-related AS events was shown by the use of a volcano plot (Figure 3(a)). The 20 most distinct OS-related AAs (Figure 3(b)), ADs (Figure 3(c)), APs (Figure 3(d)), ATs (Figure 3(e)), ESs (Figure 3(f)), MEs (Figure 3(g)), and RIs (Figure 3(h)) were shown using a bubble chart.

### 3.3. Distinction and Evaluation of AS-Based Prognostic Signature for HNSCC

**Figure 4:**
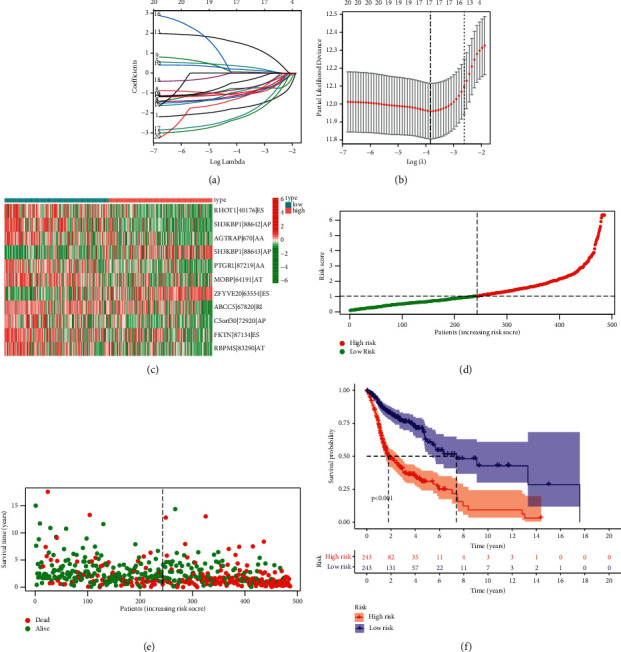
Evaluation of the performance of AS-based prognostic signature in TCGA datasets. (a) LASSO coefficient profiles. (b) LASSO deviance profiles. (c) Distribution of the AS events shown by a heat map in the TCGA dataset. (d) Distribution of risk score. (e) The survival status and duration of HNSCC patients. (f) Kaplan–Meier assays of AS-based prognostic signature in HNSCC patients.

**Figure 5:**
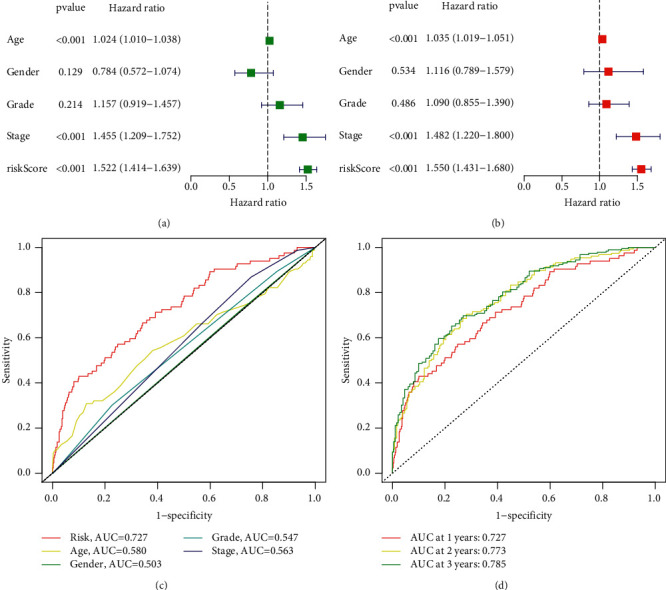
The prognostic values of AS-based prognostic signature in TCGA datasets. (a) Univariate and (b) multivariate assays in AS-based prognostic signature. (c) AUC for predicting one-year survival with different clinical features. (d) The diagnostic value of AS-based prognostic signature in predicting 1-, 2-, and 3-year survival.

**Figure 6:**
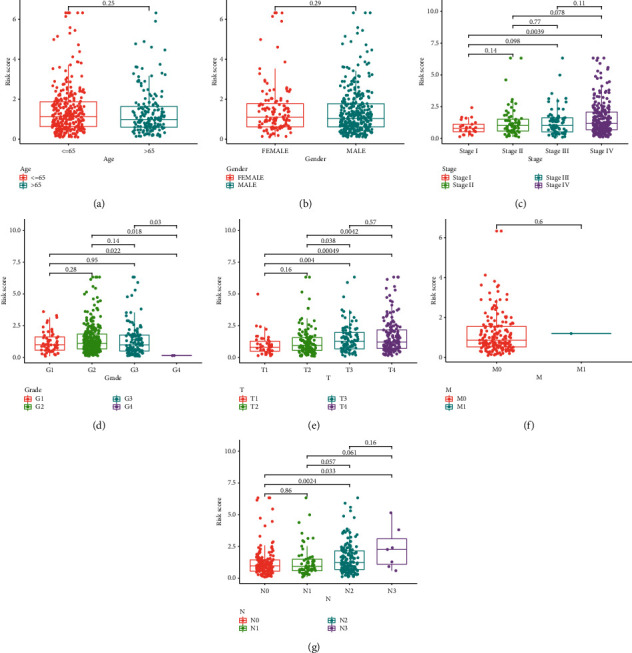
Relationship between risk score and clinical features. Distribution of risk score in (a) age, (b) gender, (c) stage, (d) grade, (e) T classification, (f) M classification, and (g) N classification.

**Figure 7:**
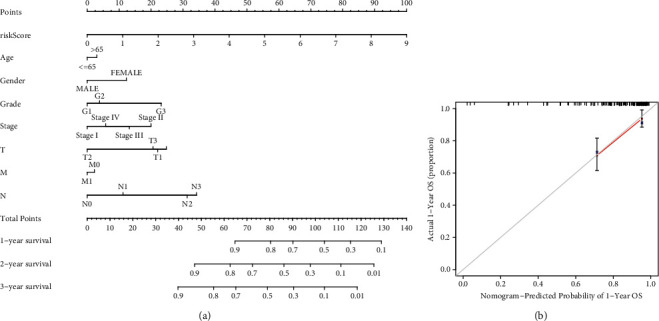
A quantitative method to predict HNSCC patients' probability of 1-, 3-, and 5-year OS. (a) Nomogram was assembled by signature and clinical stages for the prediction of HNSCC patients' survivals. (b) One-year nomogram calibration curves.

Then, a prognostic model for HNSCC patients was developed based on the abovementioned results. For avoiding overfitting, the Lasso plot and the Lambda plot were conducted (Figures 4(a) and 4(b)). Finally, 11 OS-SEs were screened for further multivariate assays. The heat map revealed that SH3KBP1|88643|AP and ZFYVE20|63554|ES might have positive effects on HNSCC, while AGTRAP|670|AA, SH3KBP1|88642|AP, RHOT1|40176|ES, PTGR1|87219|AA, MOBP|64191|AT, ABCC5|67820|RI, C5orf30|72920|AP, FKTN|87134|ES, and RBPMS|83290|AT exhibited a contrary effect (Figure 4(c)). The specimens with lower risk scores exhibited a lower risk of mortality, which were shown using the risk curve and scatterplot (Figures 4(d) and 4(e)). Then, survival assays revealed that high-risk patients showed a shorter OS than low-risk ones (Figure 4(f)). To further demonstrate the independent roles of the risk score, we performed univariate and multivariate assays and demonstrated that the system was a well-predicting model (Figures 5(a) and 5(b)). Moreover, combined with clinical variables, AUC curve analysis was performed on 1-, 2-, and 3-year OS, and the AUC value obtained by risk characteristics was the highest (Figures 5(c) and 5(d)). On the other hand, we also analyzed the clinical association of the prognostic signature based on AS in HNSCC patients, and the clinical information is shown in Figures 6(a)–6(g). Importantly, we observed that tumor specimens with advanced stages and grades exhibited a high risk score (Figures 6(c)–6(e) and 6(g)). Finally, our group constructed a prognostic nomogram using clinicopathological stage and risk score for the prediction of the clinical outcome of HNSCC patients (Figure 7(a)). The results of calibration curves exhibited an approximate diagonal, suggesting strong abilities in predicting the clinical outcome for 1-year OS using our system (Figure 7(b)).

## 4. Discussion

HNSCC remains a healthy challenge for many countries [17]. In recent years, multimodal treatments, including surgery, chemotherapy, and radiation, have improved substantially [18]. However, there was no significant increase in 5-year overall survival (OS) and no significant reduction in mortality. Identification of novel sensitive biomarkers is very important for the improvements of clinical outcome of HNSCC patients [19]. In recent years, more and more evidence indicated that misregulation of AS may result in splicing defects that are related to multiple pathological conditions including different categories of cancers, and AS events may work as potential molecular markers during the cancer diagnosis and treatment process [20, 21]. However, there are few effective prognostic biomarkers based on AS events, which may provide crucial insights into the pathobiology of HNSCC based on AS events.

In this study, many OS-related AS events were screened by using TCGA datasets. Moreover, based on the abovementioned AS events, we developed a prognostic signature that can be used to divide HNSCC patients into groups with high and low risks. Importantly, we observed that high-risk patients were correlated with a short OS. Moreover, multivariate analyses indicated that our model could be a separate prognostic factor for overall survival of HNSCC. To further explore its clinical value, we developed a nomogram model using our system and several clinical features. Importantly, the results were significant, and a strong agreement was observed. Previously, several studies have reported the prognostic value of novel models based on alternative splicing events in several types of tumors. For instance, Xie et al. developed a splicing prognostic model using AS events, which showed satisfactory predictive efficacy for the GBM patients' survival, indicating the important clinical value of AS events for the developments of novel biomarkers [22]. In uveal melanoma, 1014 AS events were recognized as prognostic AS ones in total, and a robust prognostic prediction model containing seven AS events revealed a great promise for the prediction of overall survival of patients with uveal melanoma [23]. However, the related studies in HNSCC patients were rarely reported. Our findings provided HNSCC patients with a robust prognostic signature based on AS.

However, this study has the following limitations. Firstly, we just used TCGA datasets to confirm our findings. No cross validation was applied to demonstrate our findings. Other cohorts and in vitro and in vivo assays are needed to further demonstrate this signature in the future. Secondly, it was hard to develop a suitable system by the clinical application of the AS-based prognostic signature. The high cost of sequencing chip made it hard to detect the expressions of AS events for most HNSCC patients. Thirdly, we got many AS event-related genes, but the regulation relationships among themselves and other genes were not clear. Advanced bioinformatics is needed to reveal the regulation relationship.

## 5. Conclusions

A comprehensive analysis was conducted to AS events related to overall prognostic in HNSCC, and a prognostic model was built to convincingly forecast HNSCC patients' long-term survival outcomes. These findings may contribute to ongoing efforts to develop therapeutic targets for patients with HNSCC.

## Data Availability

The analysed datasets generated during the study are available from the corresponding author on reasonable request.
